# Association between peripheral IFN-γ^+^ cytotoxic lymphocytes and response to PD-1/PD-L1-based therapy in hepatocellular carcinoma

**DOI:** 10.3389/fimmu.2026.1738116

**Published:** 2026-02-12

**Authors:** Hui Lu, Huijuan Fang, Mengqi Ruan, Zhi Duang, Yan Wang, Wenwen Liu, Qin Wang, Qiang Zhou

**Affiliations:** 1 Department of Clinical Laboratory, the Second Affiliated Hospital of Anhui Medical University, Hefei, Anhui, China; 2 Shaanxi Techshake Biotechnology, Xian, Shaanxi, China; 3 Department of Obstetrics and Gynecology, NHC Key Laboratory of Study on Abnormal Gametes and Reproductive Tract, the First Affiliated Hospital of Anhui Medical University, Hefei, Anhui, China

**Keywords:** hepatocellular carcinoma, IFN-γ, PD-1/PD-L1, peripheral cytotoxic lymphocytes, tyrosine kinase inhibitor

## Abstract

**Background:**

Programmed cell death protein 1 (PD-1)/programmed death-ligand 1 (PD-L1)-based immune checkpoint therapy (ICT), either alone or in combination with tyrosine kinase inhibitors (TKIs) or bevacizumab, benefits a subset of patients with hepatocellular carcinoma (HCC), and reliable predictive biomarkers remain limited.

**Methods:**

Between August 2024 and July 2025, 55 HCC patients treated with PD-1-based therapies were included. Objective response rate (ORR) was assessed according to the modified Response Evaluation Criteria in Solid Tumors (mRECIST). Peripheral cytotoxic lymphocyte subsets and effector functions were profiled by multiparameter flow cytometry.

**Results:**

We observed that the ICT plus TKI group exhibited a higher ORR than ICT monotherapy (54.5% vs. 29.4%; n = 22 vs. n = 17), whereas the ORR in the ICT plus bevacizumab group was comparable to ICT monotherapy (37.5% vs. 29.4%; n = 16 vs. n = 17). Compared with ICT monotherapy, patients receiving ICT plus TKI therapy had higher peripheral CD8^⁺^ cytotoxic T lymphocyte (CTL) proportions and elevated percentages of IFN-γ^+^ CTLs, natural killer (NK) cells, and natural killer T (NKT) cells (all *P* < 0.05). Across all treatment regimens, IFN-γ^+^ cytotoxic lymphocytes frequencies were associated with treatment response and showed good discrimination, whereas circulating serum IFN-γ levels were not informative.

**Conclusion:**

These findings support peripheral IFN-γ^+^ cytotoxic lymphocytes as a candidate noninvasive biomarker for stratifying HCC patients receiving PD-1/PD-L1-based therapy.

## 1 Introduction

Hepatocellular carcinoma (HCC) is the sixth most commonly diagnosed cancer and the third leading cause of cancer-related mortality worldwide (1). Most HCC patients are diagnosed at advanced stages, thereby eliminating the opportunity for surgical intervention (2). As a result, systemic therapies play a pivotal role in the clinical management of HCC. In recent years, immune checkpoint inhibitors (ICIs), particularly monoclonal antibodies targeting programmed cell death protein 1 (PD-1) or its ligand PD-L1, have reshaped the treatment landscape of HCC by restoring T-cell-mediated antitumor immunity. However, despite the approval of agents such as nivolumab, pembrolizumab, camrelizumab, tislelizumab, durvalumab, and atezolizumab, the clinical benefits of these agents remain modest. Objective response rates (ORRs) are observed in approximately 15% of patients, and only a small subset achieves durable clinical benefit in prospective phase II and III trials (3). Moreover, PD-1/PD-L1 monotherapy did not significantly improve overall survival compared with sorafenib in treatment-naive patients enrolled in phase III trials (4, 5).

To improve therapeutic outcomes, combination strategies incorporating ICIs with tyrosine kinase inhibitors (TKIs) or anti-vascular endothelial growth factor (VEGF) agents have been developed. For example, a phase II study reported a 53.6% ORR in systemic therapy-naive patients with unresectable Barcelona Clinic Liver Cancer (BCLC) stage B or C HCC treated with lenvatinib plus an anti-PD-1 antibody (6). The IMbrave150 trial further demonstrated that the combination of atezolizumab and bevacizumab significantly prolonged survival compared with sorafenib monotherapy in patients with unresectable HCC (7). More recently, a multicenter cohort study reported a 35% clinical complete response rate with atezolizumab plus bevacizumab in unresectable and transarterial chemoembolization (TACE)-unsuitable intermediate-stage HCC patients (8). Consequently, combination immunotherapies have become a key component of first-line systemic therapy for advanced HCC.

Despite these advances, the response to PD-1-based immunotherapy remains highly heterogeneous, benefiting only a subset of patients. Thus, extensive efforts have been devoted to identifying biomarkers capable of guiding treatment selection and evaluating treatment efficacy (9–11). Although PD-L1 expression has been approved by the U.S. Food and Drug Administration (FDA) as a biomarker for immunotherapy in several malignancies, its predictive value in HCC is limited. For example, PD-L1 expression does not reliably predict treatment response to pembrolizumab or atezolizumab in HCC, as noted in FDA medication guides (reference ID:5294627). In this context, there is an urgent need to identify robust and clinically applicable biomarkers to guide both initial treatment selection and dynamic monitoring in patients receiving PD-1/PD-L1-based monotherapy or combination therapies.

Cytotoxic T cells (CTLs) are well-established effectors of antitumor immunity in both preclinical and clinical settings, and their intratumoral abundance has been correlated with improved disease-free and overall survival in multiple tumor types (12). In addition to CTLs, natural killer (NK) cells and natural killer T (NKT) cells also represent key cytotoxic lymphocyte subsets with antitumor potential (13). However, most studies have focused on tumor-infiltrating lymphocytes, whereas the biomarker value of peripheral cytotoxic lymphocyte subsets remains largely unexplored in HCC.

Given the limitations of tissue sampling and the increasing interest in minimally invasive biomarkers, peripheral blood offers a more accessible and dynamic source for immune monitoring. Compared with tissue-resident lymphocytes, peripheral lymphocytes are easier to obtain and can reflect systemic immune responses. Therefore, this study aims to comprehensively evaluate the proportions and functional status of peripheral CTLs, NK cells, and NKT cells in HCC patients receiving PD-1/PD-L1-based therapy, with the goal of identifying potential peripheral immune correlates to support personalized immunotherapy strategies in HCC.

## 2 Materials and methods

### 2.1 Study design and patient data collection

A total of 55 HCC patients admitted to the Second Affiliated Hospital of Anhui Medical University between August 2024 and July 2025 were enrolled. All patients were diagnosed on the basis of the imaging findings and/or histopathological confirmation. The inclusion criteria were as follows (1): age ≥18 years (2); newly diagnosed HCC confirmed by imaging, pathology, or postoperative recurrent HCC (3); receiving PD-1/PD-L1-based immunotherapy (including monotherapy or combination with TKI or VEGF inhibitors) (4); availability of complete clinical and immunological data before and during treatment; and (5) adequate residual peripheral blood samples remaining after routine clinical laboratory testing. The exclusion criteria included the following (1): the presence of other malignancies (2); autoimmune diseases (3); concurrent treatment with TACE; or (4) incomplete medical data.

Treatment response was assessed by contrast-enhanced CT/MRI according to the modified Response Evaluation Criteria in Solid Tumors (mRECIST) for HCC (14). Complete response (CR) was defined as disappearance of any intratumoral arterial enhancement in all target lesions; partial response (PR) as at least 30% decrease in the sum of diameters of viable (arterial enhancing) target lesions relative to baseline; progressive disease (PD) as an increase of at least 20% in the sum of diameters of viable (enhancing) target lesions recorded since treatment started; and stable disease (SD) as neither PR nor PD. ORR was defined as CR plus PR. Retrospective clinical information, including sex, age, BCLC stage, Child-Pugh classification, hepatitis B/C virus (HBV/HCV) infection status, presence of cirrhosis and history of postoperative recurrence, was collected from electronic medical records. Only discarded peripheral blood samples were used in this study. Serum AFP and PIVKA-II levels were measured before initiation of PD-1/PD-L1-based therapy and at an on-treatment time point. In contrast, white blood cell counts (including lymphocytes, monocytes, and neutrophils), cytotoxic lymphocyte immunophenotyping, and functional analysis data were collected at a single on-treatment time point after therapy initiation, with the on-treatment sampling cycle varying across patients.

### 2.2 AFP and PIVKA-II detection

Serum AFP and PIVKA-II levels were measured via the ARCHITECT immunoassay kits (chemiluminescent microparticle immunoassay, Abbott, Germany; REF: 07P9077 for AFP and 01R1774 for PIVKA-II) according to the manufacturer’s instructions. All assays were performed on the Abbott ARCHITECT *i* System.

### 2.3 White cell counting

White blood cell (WBC) counts, including those of lymphocytes, neutrophils, and monocytes, were measured via an automated hematology analyzer (Mindray BC-7500, Shenzhen, China) with EDTA-anticoagulated whole blood.

### 2.4 Multicolor flow cytometry analysis

Whole blood samples were subjected to red blood cell lysis and subsequently stained for surface and intracellular markers. CD16 and CD56 were stained using antibodies conjugated to the same fluorochrome and acquired as a single combined CD16/CD56 channel. The detailed antibody information was provided in **Table 1**. Data were acquired on a BD FACSLyric flow cytometer (3-laser, 12-color, BD Biosciences, USA) and analyzed via BD FACSUITE software (v1.5, BD Biosciences).

**Table 1:** Flow cytometry antibody panel.

Target	Clone	Fluorochrome	Vendor	Application	Catalog no.
CD45	1-A3	PE-Cy7	TOIMMY BIOTECH, China	Surface	TB106006
CD3	D-A11	PerCP-Cy5.5	TOIMMY BIOTECH, China	Surface	TB106057
CD8	2-B4	APC-Cy7	TOIMMY BIOTECH, China	Surface	TB106067
CD16	4-H8	APC	TOIMMY BIOTECH, China	Surface	TB106002
CD56	H-D6	APC	TOIMMY BIOTECH, China	Surface	TB106001
Granzyme B	3-E12	FITC	TOIMMY BIOTECH, China	Intracellular	TB106004
IFN-γ	7-E11	PE	TOIMMY BIOTECH, China	Intracellular	TB106005

### 2.5 Intracellular cytokine staining

After red blood cell lysis, washed cells were resuspended in RPMI-1640 and incubated with a PMA/ionomycin/monensin stimulation cocktail (final concentrations: PMA, 81 nM; ionomycin, 1.34 µM; monensin, 2 µM; REF: TB106008, TOIMMY BIOTECH, China) together with CD16 and CD56 antibodies conjugated to the same fluorochrome. Cells were incubated at 37 °C in a 5% CO_2_ incubator for 3 h. After stimulation, cells were washed with phosphate-buffered saline (PBS) and stained with antibodies against CD45, CD3, and CD8, along with a fixable viability dye (REF: 655-0866-14, Invitrogen, USA), for 20 min at room temperature in the dark. Cells were then washed, fixed for 20 min at room temperature in the dark, and permeabilized using permeabilization buffer. Intracellular staining was performed with fluorochrome-conjugated antibodies against GZMB and IFN-γ for 40 min at room temperature in the dark. Finally, cells were washed with PBS and acquired on the flow cytometer.

### 2.6 Serum IFN-γ detection

Circulating IFN-γ was quantified using a multiplex cytokine detection kit (REF: 281501HN, Wellgrow, China) based on flow fluorescent immunoassay, according to the manufacturer’s instructions. Data acquisition was performed on a BD FACSLyric flow cytometer.

### 2.7 Correlation analysis

Correlations between immune cell functional markers and clinical parameters (AFP, PIVKA-II, and treatment response) were assessed via Spearman’s rank correlation.

### 2.8 Statistical analysis

Statistical analyses were performed using R (v4.5.1) and GraphPad Prism (v9). Two-group comparisons were performed using Student’s/Welch’s t tests or the Mann-Whitney U test, and comparisons involving more than two groups were performed using the Kruskal-Wallis test. Categorical variables were compared using the chi-square or Fisher’s exact test, as applicable. Data are presented as mean ± standard errors of the means (SEMs) or median (interquartile range), as appropriate. Given the right-skewed distributions of serum AFP and PIVKA-II, log10 transformation was applied in model-based analyses (multivariable logistic regression and the derived receiver operating characteristic (ROC) analyses) and in correlation analyses involving tumor marker changes to reduce skewness and stabilize variance. ROC curves for individual parameters were generated in GraphPad Prism. Model-based ROC curves were generated in R using the pROC package, and area under the curves (AUCs) were compared using DeLong’s test. Multivariable logistic regression was performed in R to assess independent associations with treatment response, reporting odds ratios (95% CIs) and Wald *P*-values. Covariates in multivariable logistic regression were predefined based on clinical relevance and included log10-transformed AFP, BCLC stage, Child-Pugh class, and treatment regimen. A two-sided *P* < 0.05 was considered statistically significant.

## 3 Results

### 3.1 Clinical and laboratory characteristics across treatment groups

The clinical and laboratory characteristics of patients receiving PD-1/PD-L1-based ICT alone, ICT plus TKI, or ICT plus bevacizumab were summarized in **Table 2**. There were no statistically significant differences in age, sex, etiology (HBV/HCV/nonviral), BCLC stage, cirrhosis status, Child-Pugh classification, post-resection recurrence, treatment cycles, or peripheral immune cell counts (lymphocytes, neutrophils, and monocytes) among the three groups. The serum levels of AFP and PIVKA-II were also comparable across the groups.

**Table 2:** Baseline clinical characteristics and on-treatment laboratory parameters of patients in the three treatment groups.

Factors	ICT (n=17)	ICT+TKI (n=22)	ICT+Beva (n=16)	*P* value
Age Years old, median (IQR)	59 (56–66)	71.5 (59.75-73)	59 (54–69)	0.127
Sex Male/Female	13/4	21/1	15/1	0.165
Etiology HBV/HCV/Non	12/1/4	17/1/4	9/7/0	0.961
BCLC stage B/C	5/12	4/18	0/16	0.055
Cirrhosis Yes/no	12/5	16/6	11/5	1
Child-puge A/B/C	15/2/0	17/4/1	9/7/0	0.125
Post-resection recurrence Yes/no	8/9	4/18	6/10	0.145
Treatment cycles median (IQR)	8 (4–11)	4 (2-7.5)	4.5 (3–8)	0.127
Lymphocytes x10^9^/L, median (IQR)	1.12 (0.8-1.58)	1.1 (0.85-1.66)	0.82 (00.57-1.26)	0.288
Neutrocytes x10^9^/L, median (IQR)	2.13 (1.75-2.73)	2.72 (1.62-3.45)	2.5 (1.65-2.71)	0.338
Monocytes x10^9^/L, median (IQR)	0.41 (0.26-0.58)	0.34 (0.27-0.38)	0.36 (0.26-0.43)	0.452
AFP ng/mL, median (IQR)	4 (3.08-168.4)	8.11 (3.45-81.14)	230.44 (2.07-1164.72)	0.84
PIVKA-II ng/mL, median (IQR)	30.56 (22.02-331.96)	150.74 (34.29-1468.05)	141.43 (47.84-1326.03)	0.285

Data are presented as medians with interquartile ranges (IQRs) or numbers of cases, as appropriate. *P*-values were calculated via the Kruskal-Wallis test for continuous variables and the chi-square test or Fisher’s exact test for categorical variables.

ICT, Immune checkpoint therapy; TKI, tyrosine kinase inhibitor; Beva, bevacizumab. BCLC, Barcelona Clinic Liver Cancer; Child-Pugh, Liver Function Classification; AFP, alpha-fetoprotein; PIVKA-II, protein induced by vitamin K absence or antagonist-II. AFP and PIVKA-II values shown in this table were measured at the on-treatment time point (timing varied across treatment cycles).

### 3.2 Comparison of the proportions and function of peripheral CTL, NK, and NKT cells between ICT monotherapy and ICT plus TKI/bevacizumab

Assessment of treatment outcomes revealed that the ICT plus TKI group achieved a higher ORR (54.5%, 12/22) compared to the ICT monotherapy group (29.4%, 5/17) and ICT plus bevacizumab group (37.5%, 6/16), suggesting that the addition of TKIs may enhance therapeutic efficacy. To identify potential immune correlates of treatment response, we compared the frequency and functional characteristics of peripheral cytotoxic lymphocyte subsets between patients treated with ICT alone (lower response rate) and those treated with ICT plus TKI (higher response rate). The analyzed subsets included CTLs, NK cells, and NKT cells.

**Figure 1:**
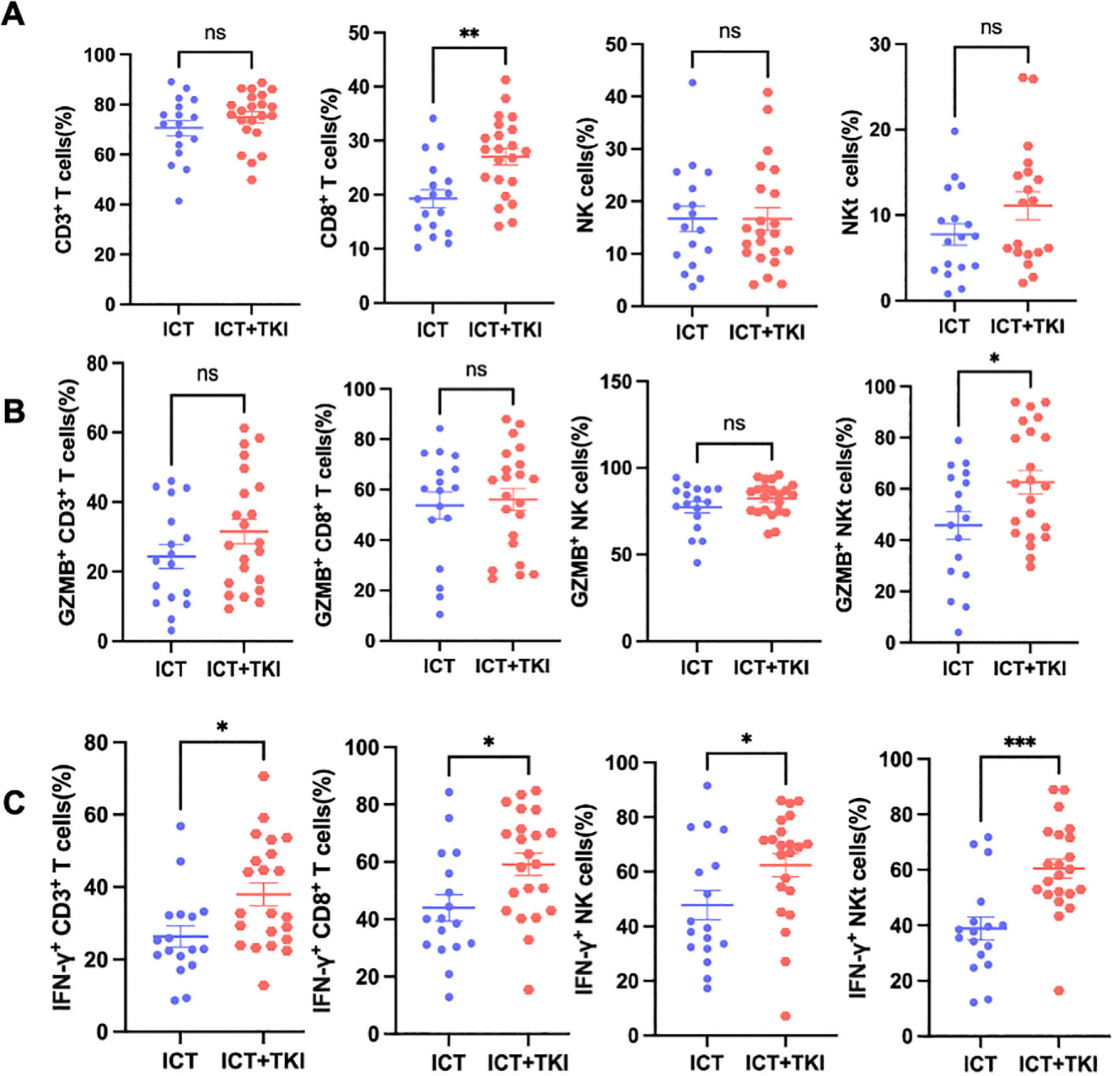
Peripheral cytotoxic lymphocyte proportions and intracellular GZMB/IFN-γ positivity in HCC patients receiving ICT alone or ICT plus TKI. **(A)** Flow cytometric analysis of the proportions of CD3^+^ T cells, CTLs, NK cells, and NKT cells within lymphocytes in HCC patients treated with ICT alone or ICT plus TKI (ICT+TKI). **(B)** Percentage of GZMB^+^ cells within CTLs, NK cells, and NKT cells. **(C)** Percentage of IFN-γ^+^ cells within CTLs, NK cells, and NKT cells. Data are presented as mean ± SEM. Statistical significance was determined using Student’s t-test or the Mann-Whitney U test. *P < 0.05; **P < 0.01; ***P < 0.001; ns: not significant.

**Figure 2:**
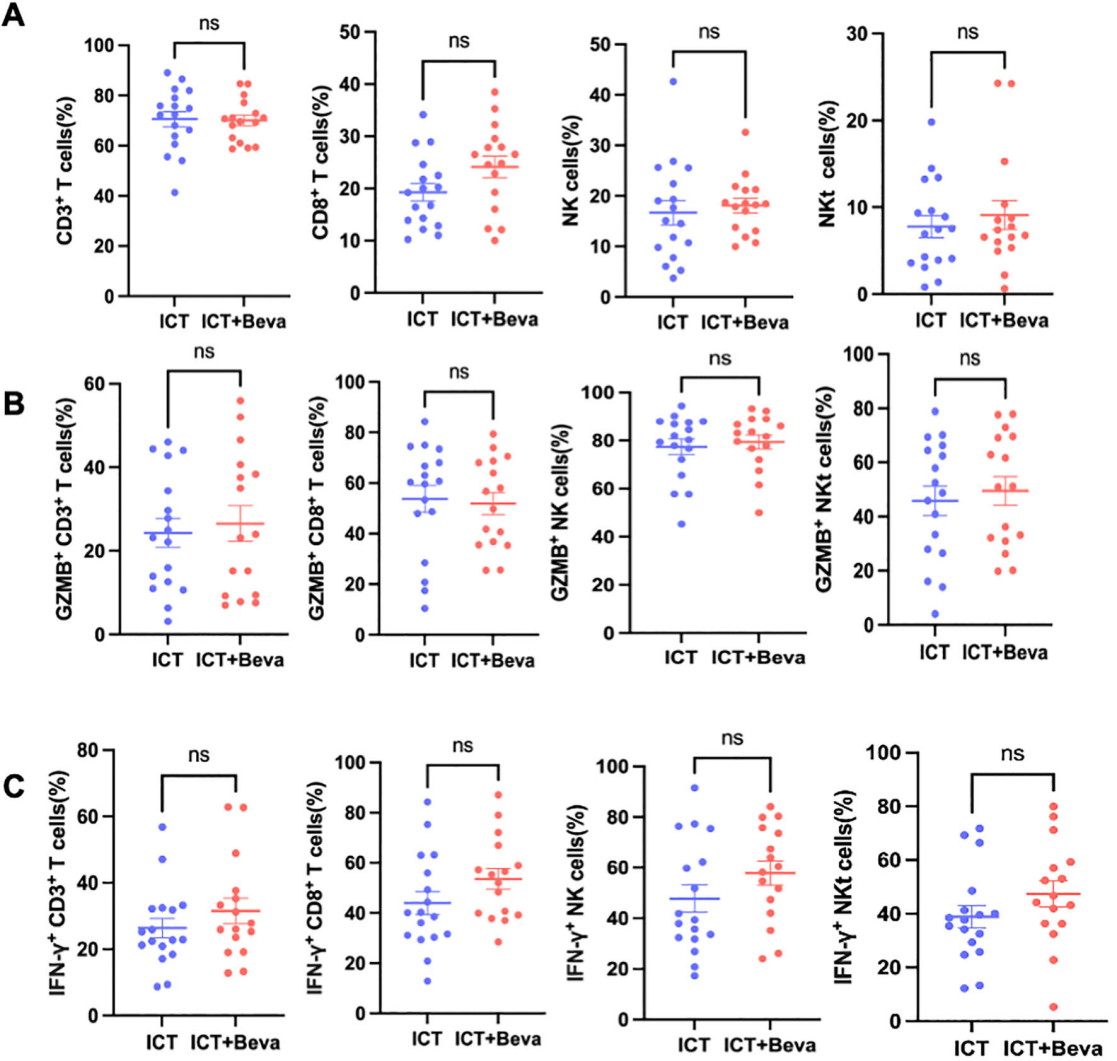
Peripheral cytotoxic lymphocyte proportions and intracellular GZMB/IFN-γ positivity in HCC patients receiving ICT alone or ICT plus bevacizumab. **(A)** Flow cytometric analysis of the proportions of CD3^+^ T cells, CTLs, NK cells, and NKT cells within lymphocytes in HCC patients treated with ICT alone or ICT plus bevacizumab (ICT+Beva). **(B)** Percentage of GZMB^+^ cells within CTLs, NK cells, and NKT cells. **(C)** Percentage of IFN-γ^+^ cells within CTLs, NK cells, and NKT cells. Data are presented as mean ± SEM. Statistical significance was determined using Student’s t-test or the Mann-Whitney U test. ns: not significant.

Detailed gating strategies for surface and intracellular cytokine staining were shown in **Supplementary Figures S1, S2**. Flow cytometric analysis demonstrated that ICT plus TKI treatment increased the proportion of CD8^+^ T cells compared with ICT monotherapy (**Figure 1A**). Functional profiling revealed that only NKT cells (CD3^+^CD16/CD56^+^) exhibited a higher percentage of GZMB^+^ cells (**Figure 1B**). In contrast, the percentage of IFN-γ^+^ cells was elevated in ICT plus TKI group across all cytotoxic lymphocyte subsets, including CTLs, NK cells, and NKT cells (**Figure 1C**). No differences were observed between the ICT monotherapy and ICT plus bevacizumab groups in either proportions or functional status of cytotoxic lymphocytes, which is consistent with their comparable ORR (**Figures 2A–C**).

### 3.3 Comparison of proportions and function of peripheral CTL, NK, and NKT cells between responders and non-responders

**Figure 3:**
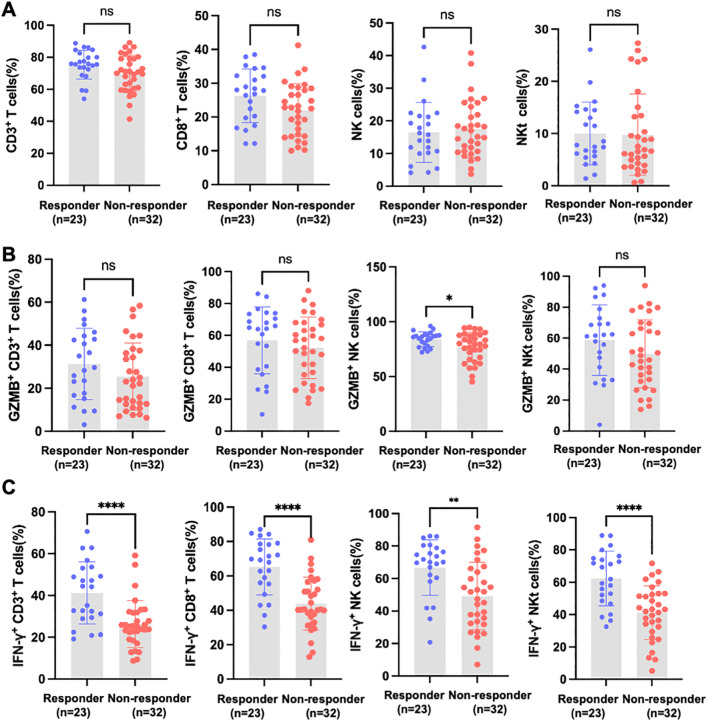
The functional activation of peripheral cytotoxic lymphocytes distinguishes responders from non-responders to PD-1/PD-L1-based therapy. **(A)** Flow cytometric analysis of the proportions of CD3^+^ T cells, CTLs, NK cells, and NKT cells within lymphocytes in HCC patients classified as responders (n = 23) or non-responders (n = 32) to PD-1-based immunotherapy. **(B)** Percentage of GZMB^+^ cells within CTLs, NK cells, and NKT cells. **(C)** Percentage of IFN-γ^+^ cells within CTLs, NK cells, and NKT cells. The data are presented as the mean ± SEM. Statistical significance was determined via Student’s *t*-test or the Mann-Whitney U test. *P < 0.05; **P < 0.01; ****P < 0.0001; ns: not significant.

To further investigate the association between peripheral immune status and treatment response to PD-1/PD-L1-based therapy in HCC patients, patients were stratified into responders and non-responders on the basis of ORR. No significant differences were observed in the proportions of these subsets between responders and non-responders (**Figure 3A**).

We next assessed the functional status of cytotoxic lymphocytes by quantifying the percentage of GZMB^+^ and IFN-γ^+^ cells. Among the three cytotoxic lymphocyte subsets, only NK cells showed a significantly higher percentage of GZMB^+^ cells in responders compared with non-responders (**Figure 3B**). In contrast, the percentage of IFN-γ^+^ cells was markedly higher in responders across all cytotoxic lymphocyte subsets, including CD8^+^ T cells, NK cells, and NKT cells (**Figure 3C**).

To evaluate whether the clinical and laboratory characteristics of these patients are associated with treatment response in HCC patients, we compared baseline clinical characteristics and peripheral blood parameters between responders and non-responders. As shown in **Table 3**, the serum AFP and PIVKA-II levels were significantly lower in responders than in non-responders. Although there was a modest difference in age, no significant differences were observed in sex, etiology (HBV/HCV/nonviral), BCLC stage, cirrhosis status, Child-Pugh classification, post-resection recurrence, treatment cycles, or peripheral immune cell counts (lymphocytes, neutrophils, and monocytes) between the two groups.

**Table 3:** Baseline clinical characteristics and on-treatment laboratory parameters of PD-1 based therapy responders and non-responders.

Factors	Responder (n=23)	Non-responder (n=32)	*P* value
Age years old, median (IQR)	69 (59–73)	59 (53–68)	0.03
Sex Male/Female	22/1	27/5	0.38
Etiology HBV/HCV/Non	16/0/7	26/3/3	0.07
BCLC stage B/C	4/19	5/27	1.00
Cirrhosis Yes/no	13/10	26/6	0.09
Child-puge A/B/C	19/3/1	22/10/0	0.11
Post-resection recurrence Yes/no	6/17	12/20	0.55
Treatment cycles median (IQR)	5 (3-8)	4 (2.75-10.25)	0.904
Lymphocytes x10^9^/L, median (IQR)	1.09 (0.83-1.62)	0.99 (0.57-1.31)	0.24
Neutrocytes x10^9^/L, median (IQR)	2.48 (1.67-2.76)	2.535 (1.69-3.44)	0.82
Monocytes x10^9^/L, median (IQR)	0.33 (0.28-0.41)	0.38 (0.27-0.49)	0.28
AFP ng/mL, median (IQR)	3.79 (2.62-17.06)	130.535 (3.34-945.75)	0.01
PIVKA.II ng/mL, median (IQR)	30.56 (23.91-77.70)	337.48 (105.96-4235.45)	0.0002

Data are presented as medians with interquartile ranges (IQRs) or numbers of cases, as appropriate. *P*-values were calculated via the Wilcoxon test or t-test for continuous variables and the chi-square test or Fisher’s exact test for categorical variables. AFP and PIVKA-II values shown in this table were measured at the on-treatment time point (timing varied across treatment cycles).

### 3.4 IFN-γ positive cytotoxic lymphocyte subsets correlate with treatment response and tumor marker declines in HCC

**Figure 4:**
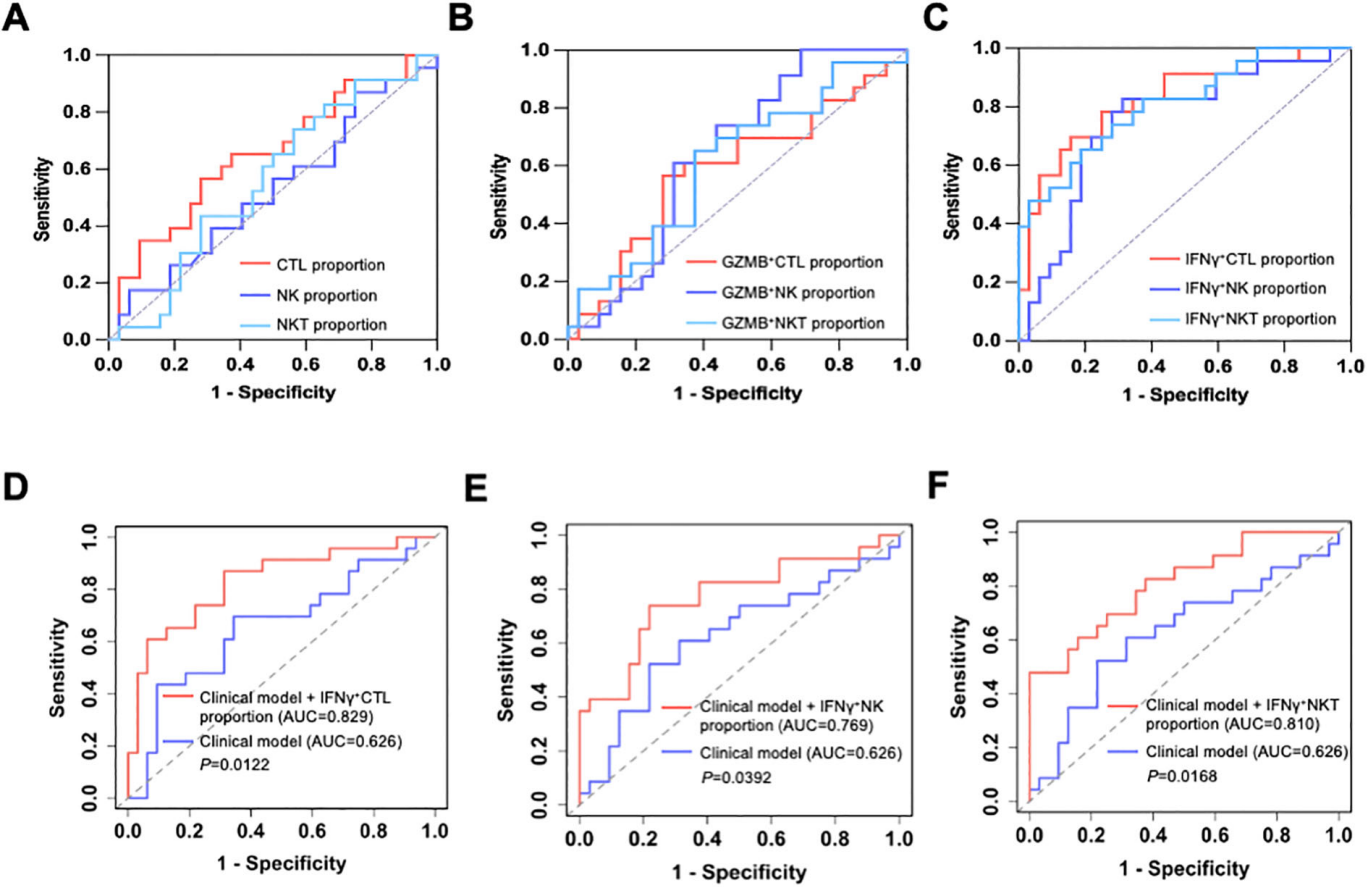
ROC curve analysis of peripheral cytotoxic lymphocyte proportions, functional markers, and multivariable clinical models for discriminating the response to PD-1/PD-L1-based therapy in HCC patients. **(A)** ROC curves for the proportions of CTL, NK, and NKT cells within lymphocytes. **(B)** ROC curves for the proportion of GZMB^+^ CTLs, NK cells, and NKT cells. **(C)** ROC curves for the proportion of IFN-γ^+^ cells within CTLs, NK cells, and NKT cells. **(D-F)** ROC curves comparing a clinical model with an extended model incorporating percentages of IFN-γ^+^ cytotoxic lymphocytes. The clinical model included AFP (log-transformed), BCLC stage (B vs C), baseline liver function (Child-Pugh A vs B/C), and treatment regimen (ICT, ICT+TKI, ICT+Beva). The extended models additionally included the percentage of IFN-γ^+^ CTL **(D)**, NK **(E)**, or NKT **(F)**, respectively. The diagonal dashed line represents the line of no discrimination (AUC = 0.5).

To evaluate the discriminative performance of peripheral cytotoxic lymphocyte measurements for treatment response, we performed ROC analysis. In single-parameter ROC curves, the proportions of CTLs, NK cells, and NKT cells (**Figure 4A**), as well as GZMB^+^ (**Figure 4B**) and IFN-γ^+^ functional subsets (**Figure 4C**), showed varying degrees of discrimination between responders and non-responders, with IFN-γ^+^ subsets exhibiting the most favorable curve patterns. The specific AUC values and P values were summarized in **Table 4**. For model-based ROC analyses, the clinical model yielded an AUC of 0.626. Incorporating the frequencies of IFN-γ^+^ CTL, IFN-γ^+^ NK, or IFN-γ^+^ NKT to the clinical model increased the AUC to 0.829, 0.769, and 0.810, respectively (**Figures 4D–F**). AUC comparisons using DeLong’s test indicated significant improvements for all three augmented models (*P* = 0.0122, 0.0392, and 0.0168, respectively).

**Table 4:** AUCs and associated P values for peripheral cytotoxic lymphocyte proportions and functional markers for treatment response discrimination.

Group	CTL%	NK%	NKT%	GZMB+ CTL%	GZMB+ NK%	GZMB+ NKT%	IFNγ+ CTL%	IFNγ+ NK%	IFNγ+ NKT%
AUC	0.65	0.53	0.56	0.59	0.64	0.61	0.82	0.75	0.81
*P value*	0.06	0.75	0.46	0.28	0.07	0.17	<0.0001	0.002	0.0001

In multivariable logistic regression adjusting for AFP (log10-transformed), Child-Pugh class, BCLC stage, and treatment regimen, higher percentage of IFN-γ^+^ cytotoxic lymphocytes remained independently associated with treatment response: IFN-γ^+^ CTL% (adjusted OR = 1.083, 95% CI 1.035-1.134; *P* = 0.0006), IFN-γ^+^ NK% (OR = 1.049, 95% CI 1.012-1.086; *P* = 0.0089), and IFN-γ^+^ NKT% (OR = 1.086, 95% CI 1.033-1.143; *P* = 0.0013) (**Table 5**).

**Table 5:** Multivariable logistic regression for objective response (adjusted for AFP, Child-Pugh, BCLC, treatment regimen).

Items	Adjusted OR	95% CI	*P* value
IFNγ^+^CTL%	1.083	1.035-1.134	<0.001
IFNγ^+^NK%	1.049	1.012-1.086	0.009
IFNγ^+^NKT%	1.086	1.033-1.142	0.001

Adjusted OR, adjusted odds ratio; CI, confidence interval. Odds ratios are reported per 1%-point increase in the indicated immune variable and were adjusted for AFP (log10-transformed), BCLC stage, Child-Pugh class, and treatment regimen.

**Figure 5:**
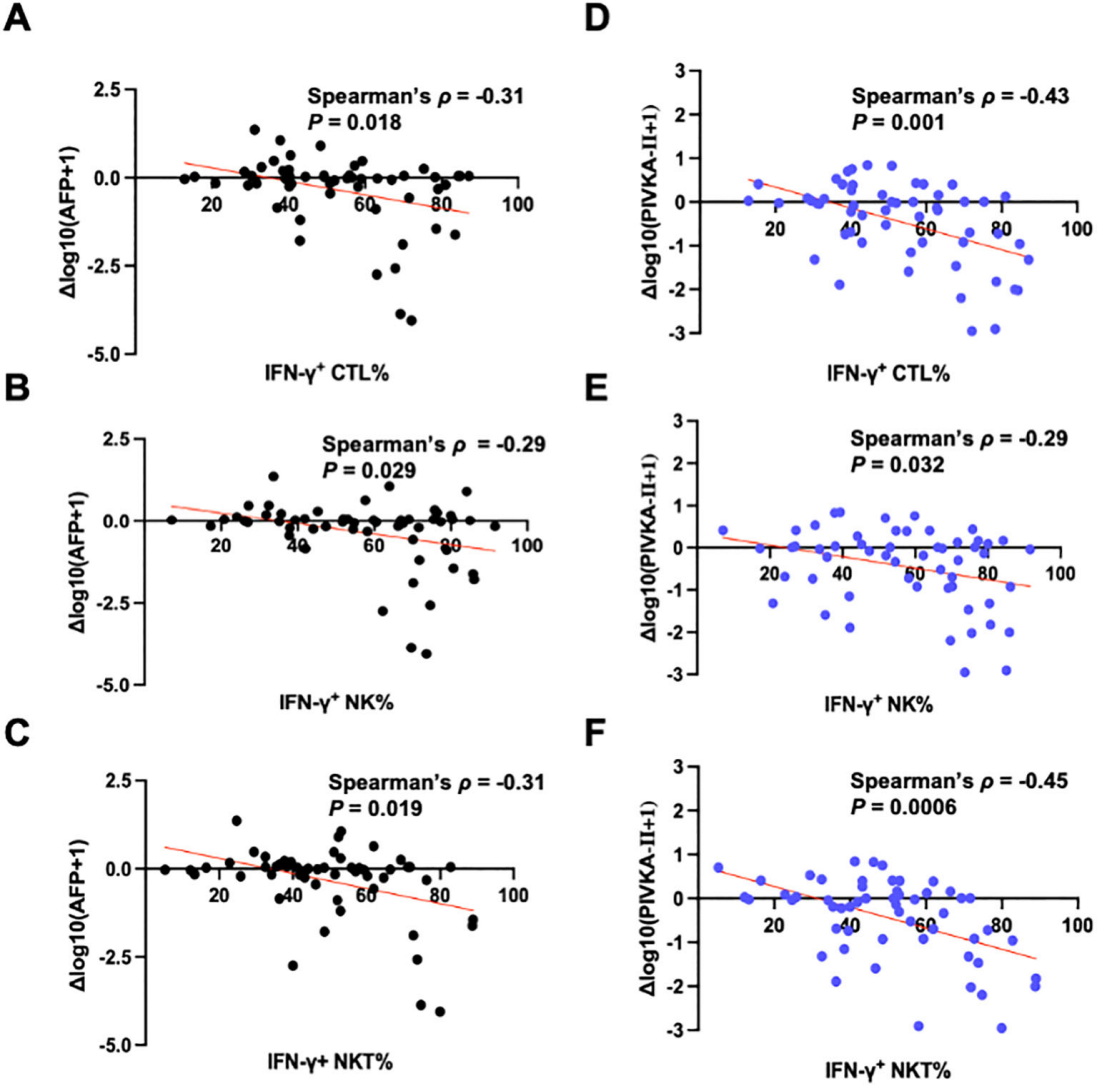
IFN-γ^+^ cytotoxic lymphocytes are inversely correlated with serum tumor marker dynamics. **(A-C)** Correlations between the percentage of IFN-γ^+^ cells within CTLs, IFN-γ^+^ NK cells, and IFN-γ^+^ NKT cells and Δlog10(AFP + 1), defined as log10(AFP_post+1) − log10(AFP_pre+1). **(D-F)** Correlations between the same IFN-γ^+^ cytotoxic lymphocyte subsets and Δlog10(PIVKA-II+1), defined as log10(PIVKA-II_post+1) − log10(PIVKA-II_pre+1). Spearman correlation coefficients (*ρ*) and *P*-values are indicated. The red lines indicate linear fits for visualization only.

We then examined the association between IFN-γ^+^ cytotoxic lymphocyte subsets (CTL, NK, and NKT) and tumor marker dynamics. The results showed that higher percentages of IFN-γ^+^ cytotoxic lymphocyte subsets were significantly associated with declines in AFP and PIVKA-II during treatment, as indicated by more negative Δlog10(AFP + 1) (**Figures 5A–C**) and Δlog10(PIVKA-II+1) (**Figures 5D–F**) values.

### 3.5 Circulating IFN-γ levels are not associated with response to PD-1/PD-L1-based therapy

**Figure 6:**
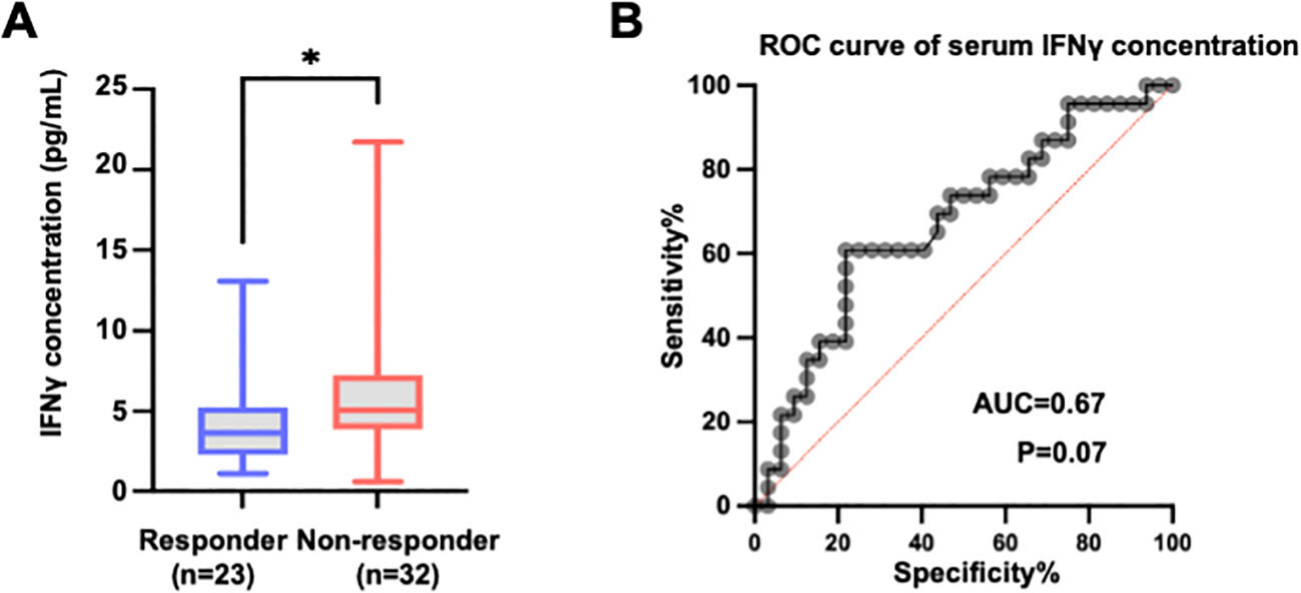
The circulating IFN-γ concentration is elevated in non-responders and has a limited ability to discriminate treatment response. **(A)** Comparison of serum IFN-γ concentrations between responders (n = 23) and non-responders (n = 32) to PD-1/PD-L1-based therapy. IFN-γ levels were significantly higher in non-responders than in non-responders. The data are presented as medians with interquartile ranges (IQRs) and were visualized via box-and-whisker plots. Statistical significance was determined via the Mann-Whitney U test. **(B)** ROC curve analysis evaluating the ability of the baseline serum IFN-γ concentration to distinguish responders from non-responders. The area under the curve (AUC) and *P*-values are indicated. The red diagonal line represents the line of no discrimination (AUC = 0.5).

Given the strong association between the percentage of IFN-γ^+^ cytotoxic lymphocytes and the treatment response, we next evaluated whether circulating IFN-γ levels might similarly serve as a potential biomarker. Unexpectedly, baseline serum IFN-γ concentrations were lower in responders than in non-responders (*P < 0.05*), in contrast to the elevated percentage of IFN-γ^+^ cytotoxic lymphocytes from the same patients (**Figure 6A**). ROC analysis of serum IFN-γ yielded an AUC of 0.67 with a P value of 0.07 for discriminating responders from non-responders (**Figure 6B**), indicating limited discriminatory power.

## 4 Discussion

PD-1/PD-L1-based therapies, including ICT alone, ICT combined with TKIs, or ICT combined with bevacizumab, are now widely used in clinical practice and form the foundation of multiple first-line regimens (15, 16). These therapeutic approaches have transitioned the management of HCC into the immunotherapy era. However, a considerable proportion of HCC patients fail to respond to these therapies, underscoring the urgent need for robust biomarkers that can predict therapeutic response and facilitate personalized treatment selection.

Although the FDA has approved several biomarkers to predict the efficacy of immune checkpoint blockade in other cancer types, including PD-L1 expression, tumor gene mutation burden (TMB), deficient mismatch repair (dMMR), and microsatellite instability-high (MSI-H) (17), these indicators are not applicable to HCC in most cases. HCC typically has a low TMB and rarely displays dMMR or MSI-H status (18, 19). Furthermore, the application of these biomarkers requires access to tumor tissue, which is often challenging in HCC, given the difficulty and heterogeneity of tissue biopsy. Therefore, there is a critical need to develop biomarkers that are accurate, minimally invasive, and suitable for routine clinical use. Ideally, such biomarkers should be measurable in peripheral blood.

In this study, we systematically analyzed the peripheral immune characteristics of HCC patients receiving PD-1/PD-L1-based therapy. We focused on cytotoxic lymphocyte subsets, including CD8^+^ T cells, NK cells, and NKT cells, by evaluating both their frequency and functional capacity. Our results revealed that patients treated with ICT plus TKI therapy presented a higher proportion of peripheral CD8^+^ T cells than ICT monotherapy. This result suggested that TKI therapy increased the proportion of peripheral CD8^+^ T cells, which is consistent with previous studies showing that combination treatment with ICT and TKIs increased CD8^+^ T-cell infiltration (6, 20). However, we observed that treatment responsiveness was more strongly associated with the functional state of these cells, particularly their ability to produce IFN-γ. Among all cytotoxic subsets, the frequencies of IFN-γ positive CD8^+^ T cells and NKT cells were most strongly correlated with the clinical response. Moreover, the observed association between IFN-γ^+^ cytotoxic lymphocyte subsets (CTLs, NK cells, and NKT cells) and tumor marker dynamics suggests that IFN-γ^+^ cytotoxic lymphocytes are linked not only to treatment response but also to on-treatment tumor regression, particularly as reflected by changes in PIVKA-II levels.

Despite the increased percentages of IFN-γ^+^ cytotoxic lymphocytes in responders, we found that serum IFN-γ concentrations were paradoxically lower in responders. This discrepancy may reflect differences in the source and biological context of IFN-γ production. Unlike intracellular measurements that directly assess the functional status of tumor-reactive T and NK cells, serum IFN-γ levels represent a composite output from various immune and nonimmune sources, including exhausted T cells, regulatory populations, myeloid cells, and inflamed tissues. Elevated serum IFN-γ levels in non-responders are likely attributed to chronic immune activation or compensatory inflammation, rather than to effective cytotoxic immune engagement. Moreover, high serum IFN-γ levels are associated with immune dysfunction in some cancer contexts, including T-cell exhaustion, impaired effector function, and increased immunosuppressive signaling through PD-L1 upregulation (21). These findings underscore the limitations of relying solely on systemic cytokine levels to assess antitumor immunity.

Our findings underscore the clinical potential association of peripheral cytotoxic lymphocyte subsets with therapeutic outcomes in HCC patients. Although tumor-infiltrating lymphocytes (TILs) are direct effectors within the tumor microenvironment, obtaining them requires invasive procedures and is often limited by tissue heterogeneity. Compared with tissue-resident lymphocytes, peripheral lymphocytes are more readily accessible and therefore represent a more practical source of immune biomarkers for evaluating patient responses to therapy (22). This advantage not only enables dynamic, minimally invasive monitoring during treatment but also positions peripheral blood immune profiling as a viable and noninvasive approach for guiding patient stratification and optimizing therapeutic decisions.

Several limitations warrant discussion. First, the sample size was relatively small and included patients undergoing different combination regimens, which could introduce variability. Second, although we identified strong associations between intracellular IFN-γ expression and treatment response, the underlying mechanistic basis of this observation was not explored and warrants further investigation. Increased IFN-γ expression may reflect enhanced antigen recognition, improved costimulatory signaling, or reduced immune suppression. Moreover, due to the retrospective, real-world nature of the cohort and the limited availability of paired baseline samples, all parameters were analyzed at a single on-treatment time point and the timing of on-treatment blood sampling varied among patients, as samples were collected at different treatment cycles according to routine clinical practice. Future prospective studies with longitudinal immune monitoring will be required to further elucidate the temporal evolution of antitumor immune responses during PD-1/PD-L1-based therapy. Finally, the association between peripheral IFN-γ^+^ cytotoxic lymphocytes and response to PD-1/PD-L1-based therapy requires validation in independent cohorts before it can be applied clinically.

In conclusion, functional immune profiling of peripheral cytotoxic lymphocytes, particularly IFN-γ production, provides a more precise reflection of tumor-specific immune competence and correlates more closely with clinical benefit. Our results reveal that integrating these cellular immune assays with traditional clinical and biochemical indicators may improve patient stratification and help guide immunotherapeutic decision-making.

## Data Availability

The original contributions presented in the study are included in the article/**Supplementary Material**. Further inquiries can be directed to the corresponding author.

## Supplementary material

Supplementary Figure 1 Gating strategy for surface phenotyping of peripheral cytotoxic lymphocyte subsets. Representative flow cytometry plots showing the sequential gating strategy. Forward scatter area (FSC-A) vs. side scatter area (SSC-A) were used to gate lymphocyte-sized cells (P1). Forward scatter height (FSC-H) vs. forward scatter area (FSC-A) was used to separate single cells from aggregates. Lymphocytes were further selected based on CD45 expression (CD45^+^ lymphocyte gate). CD3^+^ T cells were identified within the lymphocyte gate. Cytotoxic lymphocyte subsets were then defined based on CD3, CD8, and CD16/CD56 expression: CTLs were defined as CD3^+^CD8^+^ cells; NK cells as CD3^+^CD16/CD56^+^ cells; and NKT cells as CD3^+^ CD16/56^+^ cells.

Supplementary Figure 2 Gating strategy for intracellular cytokine staining to define GZMB^+^ and IFN-γ^+^ cells in peripheral cytotoxic lymphocyte subsets. Representative flow cytometry plots showing the sequential gating strategy. FSC-A vs. s SSC-A were used to exclude debris. Lymphocytes were further selected based on CD45 expression (CD45^+^ lymphocyte gate). Live cells were identified by excluding FVD-positive events within CD45^+^ lymphocytes. CD3^+^ T cells, CTLs (CD3^+^CD8^+^), NK cells (CD3^+^CD16/CD56^+^), and NKT cells (CD3^+^ CD16/56^+^) were gated from live CD45^+^ lymphocytes respectively. IFN-γ^+^ and GZMB^+^ subsets were quantified within each population.
