# Supplementary figures and images for: Association between peripheral IFN-γ+ cytotoxic lymphocytes and response to PD-1/PD-L1-based therapy in hepatocellular carcinoma
Source: Front Immunol. 2026 Feb 12;17:1738116. doi: 10.3389/fimmu.2026.1738116 (PMC12935948; doi:10.3389/fimmu.2026.1738116)

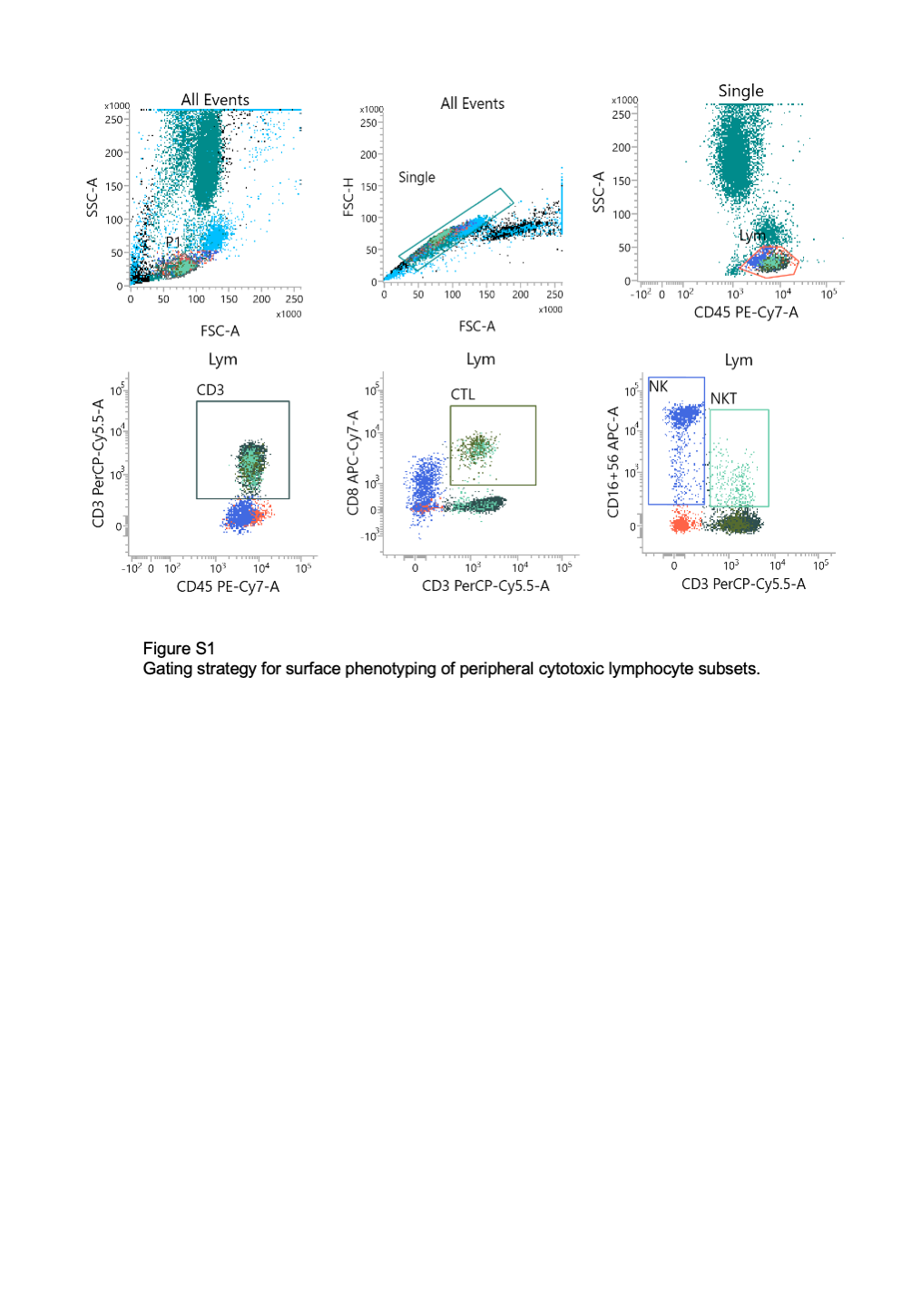

Supplement: Supplementary Figure 1 — Gating strategy for surface phenotyping of peripheral cytotoxic lymphocyte subsets. Representative flow cytometry plots showing the sequential gating strategy. Forward scatter area (FSC-A) vs. side scatter area (SSC-A) were used to gate lymphocyte-sized cells (P1). Forward scatter height (FSC-H) vs. forward scatter area (FSC-A) was used to separate single cells from aggregates. Lymphocytes were further selected based on CD45 expression (CD45+ lymphocyte gate). CD3+ T cells were identified within the lymphocyte gate. Cytotoxic lymphocyte subsets were then defined based on CD3, CD8, and CD16/CD56 expression: CTLs were defined as CD3+CD8+ cells; NK cells as CD3+CD16/CD56+ cells; and NKT cells as CD3+ CD16/56+ cells. [file Image1.tiff]

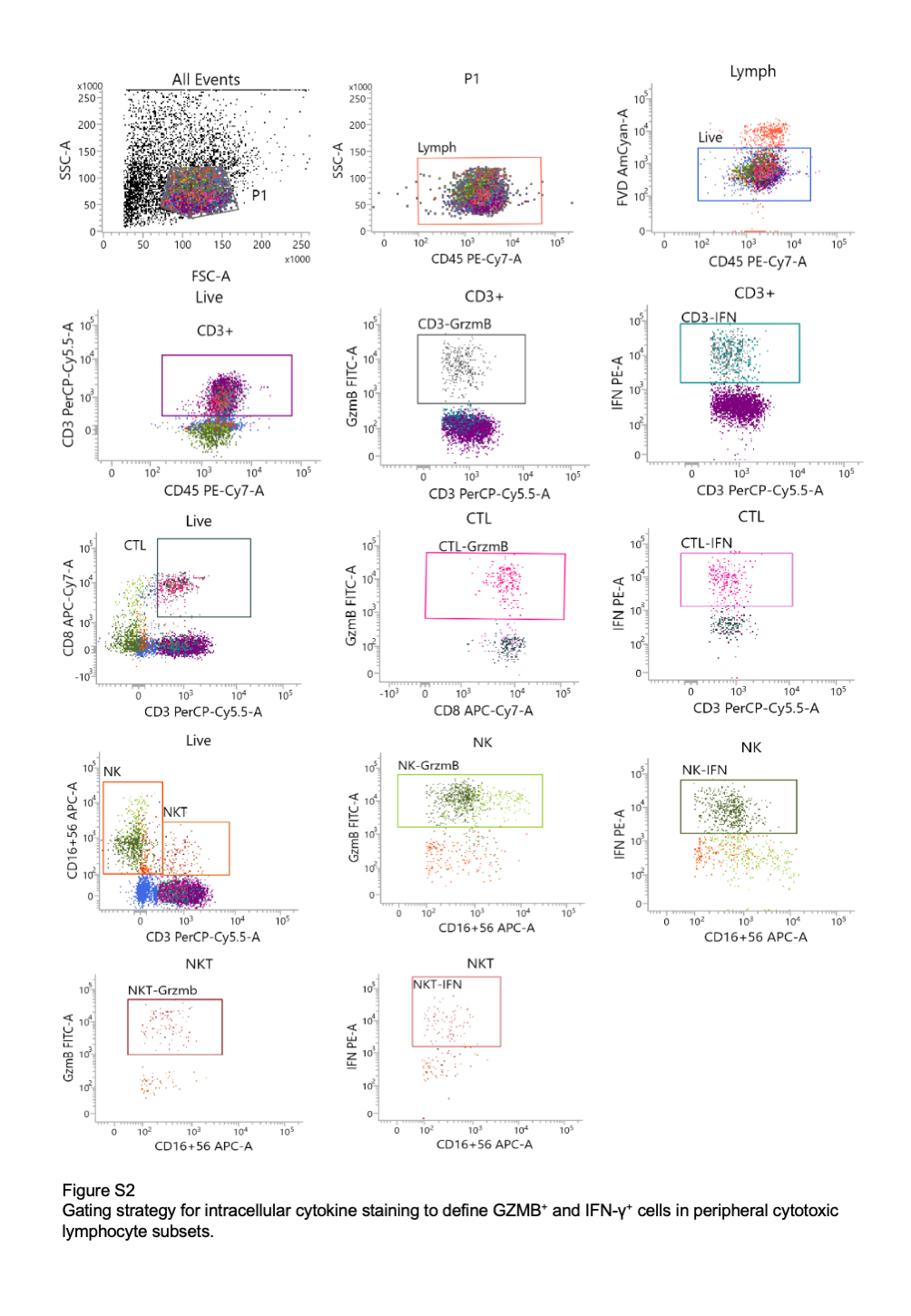

Supplement: Supplementary Figure 2 — Gating strategy for intracellular cytokine staining to define GZMB+ and IFN-γ+ cells in peripheral cytotoxic lymphocyte subsets. Representative flow cytometry plots showing the sequential gating strategy. FSC-A vs. s SSC-A were used to exclude debris. Lymphocytes were further selected based on CD45 expression (CD45+ lymphocyte gate). Live cells were identified by excluding FVD-positive events within CD45+ lymphocytes. CD3+ T cells, CTLs (CD3+CD8+), NK cells (CD3+CD16/CD56+), and NKT cells (CD3+ CD16/56+) were gated from live CD45+ lymphocytes respectively. IFN-γ+ and GZMB+ subsets were quantified within each population. [file Image2.tiff]
